# Supplementary material for: Mediating and Moderating Mechanisms in the Relationship Between Social Media Use and Adolescent Aggression: A Scoping Review of Quantitative Evidence
Source: Eur J Investig Health Psychol Educ. 2025 Jun 3;15(6):98. doi: 10.3390/ejihpe15060098 (PMC12192510; doi:10.3390/ejihpe15060098)
Supplement: Supplementary file 1 [file ejihpe-15-00098-s001.zip › Supplementary Table S1.pdf]

Table S1. Summary of included studies: mediators, moderators, and key findings

Author(s), Year	Country	Sample (Age, N)	Study design	Social media variable(s)	Aggression type(s)	Mediator(s)	Moderator(s)	Key findings
Achuthan et al., 2022	India & Singapore	Adolescents (14–19, N=522) and Parents (N=234)	Cross-sectional, SEM (TPB framework)	Instagram use, cyber behavior scales (CB, CV, BS)	Cyberbullying, Cybervictimization, Bystander behavior	Parental behavior constructs (Attitude, Perceived Control, Subjective Norms)	Parent gender, Adolescent gender, Culture (India vs. Singapore)	Parental perceived control and attitude significantly predicted adolescent cyber behavior; gender of both parents and adolescents moderated associations; Indian youth showed more cyberbullying, Singaporean youth more victimization and prosocial bystander behavior.
Aljasir & Alsebaei, 2022	Saudi Arabia	Adolescents (12–17, N=387)	Cross-sectional survey	Cyberbullying and cybervictimization on social media	Cyberbullying perpetration and victimization	None	Age and gender (3-way interaction)	Lower use of parental mediation strategies (active, restrictive, monitoring, safety) predicted higher cyberbullying and victimization. Restrictive and internet safety strategies were most protective. Age and gender moderated these effects.
Alsebaei & Aljasir, 2024	Saudi Arabia	Adolescents (12–17, N=389 dyads with parents)	Cross-sectional,	Social media use, parental mediation strategies	Cyberbullying, Cybervictimization	None tested	Parents' age, gender, education level	Active, restrictive, and safety mediation reduce aggression; technical

Table S1. Summary of included studies: mediators, moderators, and key findings

Author(s), Year	Country	Sample (Age, N)	Study design	Social media variable(s)	Aggression type(s)	Mediator(s)	Moderator(s)	Key findings
			Regression + Moderation					mediation increases it. Age and education moderated these effects; gender did not.
Bakan Kıraç et al., 2024	Turkey	Adolescents (12–14, N=406)	Cross-sectional, Mediation (PROCESS Macro)	Internet addiction (generalized, likely includes social media use)	Anger control (linked to aggression tendencies)	Psychological resilience	None tested	Internet addiction negatively predicted both anger control and psychological resilience; resilience partially mediated the relationship. Higher resilience reduced the impact of internet addiction on anger dysregulation.
Bedrosova et al., 2025	Czech Republic	Adolescents (13–18, N=658)	Experimental (between-subjects), SEM	Instagram cyberhate scenarios	Weight-based cyberhate (verbal aggression)	None tested	Anti-fat attitudes, Gender	Victim blaming was higher for plus-size victims, moderated by anti-fat attitudes and gender (boys blamed more). No significant effect on minimizing consequences. Body-positive content had no moderating effect.
Cebollero-Salinas et al., 2022	Spain	Adolescents (12–18, N=1013)	Cross-sectional, SEM	Cybergossip, Problematic Internet Use (PIU)	Cyberaggression, Cybervictimization	None tested	Gender, Age group, Age at	Cybergossip and PIU significantly predict cyberaggression and

Table S1. Summary of included studies: mediators, moderators, and key findings

Author(s), Year	Country	Sample (Age, N)	Study design	Social media variable(s)	Aggression type(s)	Mediator(s)	Moderator(s)	Key findings
							first smartphone ownership	victimization. Cybergossip's effect on aggression is stronger in girls and younger adolescents; PIU's effect on victimization is stronger in boys and younger adolescents.
Q. Chen et al., 2025	China (Public + Vocational Schools)	Adolescents (13–18, N=1,716)	Cross-sectional, SEM	Cyberbullying via social media and messaging apps	Cyberbullying victimization, Cyberbully-victim dual role	Online social capital, Internet self-efficacy	School type (public vs. vocational, in stratified SEM models)	Internet self-efficacy and online social capital mediate cyberbullying's effect on mental health (anxiety, depression, stress); mediation paths differ by school type: social capital only significant in public school sample, Internet self-efficacy significant in both.
Colella et al., 2024	Italy	Preadolescents, ages 10–14, N = 489	Cross-sectional, quantitative	Problematic Social Media Use (PSMU)	Cyberbullying, Cybervictimization	Moral Disengagement	Retaliation	PSMU was positively associated with both cyberbullying and cybervictimization. Moral disengagement mediated this relationship. Retaliation moderated both paths:

Table S1. Summary of included studies: mediators, moderators, and key findings

Author(s), Year	Country	Sample (Age, N)	Study design	Social media variable(s)	Aggression type(s)	Mediator(s)	Moderator(s)	Key findings
								higher retaliation strengthened the links between PSMU and aggression outcomes.
Colella et al., 2025	Italy & Spain	Preadolescents (9–14, N=895)	Cross-sectional, Serial Mediation (PROCESS Model 6)	Problematic Social Media Use (BSMAS scale)	Cyberbullying perpetration (ECIP-Q)	Problematic Social Media Use, Moral Disengagement	Country (included as covariate)	Cybervictimization positively predicts cyberbullying; PSMU and MD serially mediate this link. PSMU predicts MD, which in turn predicts CB. Effects are robust across both countries. Strong support for General Aggression Model.
Doğrusever & Bilgin, 2025	Turkey	Adolescents (14–18, N=2047)	Cross-sectional, Serial Mediation (PROCESS)	Problematic Internet Use (PIU)	Hostility (verbal aggression, frustration, etc.)	Hostility, Depression (both serial and individual)	None tested	Family support negatively predicts PIU; hostility and depression mediate this link. Hostility is a stronger mediator than depression; low family support increases hostility → depression → PIU.
Ferreira et al., 2021	Portugal	Adolescents (11–18, N=676)	Cross-sectional, SEM	Cyberbullying observation (on social	Online verbal aggression (self-	Social relationships with victim/bystanders,	Gender (exploratory)	Observing cyberbullying increased verbal aggression; social ties

Table S1. Summary of included studies: mediators, moderators, and key findings

Author(s), Year	Country	Sample (Age, N)	Study design	Social media variable(s)	Aggression type(s)	Mediator(s)	Moderator(s)	Key findings
				media, games, chat apps)	reported use by bystanders)	Self-efficacy to solve cyberbullying		and self-efficacy mediated the effect; boys reported more verbal aggression; girls higher self-efficacy. Mediation effects were weaker than direct effects.
Geng et al., 2021	China	Adolescents (10–16, N=941)	Cross-sectional, Moderated Mediation (SEM + bootstrapping)	Envy on SNS (WeChat, Qzone, TikTok), social comparison orientation (SCO)	Cyberbullying perpetration and victimization	Envy on SNS	Body satisfaction	SCO predicts cyberbullying perpetration and victimization via envy; higher body satisfaction weakens both direct (SCO → envy) and indirect (SCO → envy → cyberbullying) paths. Strong statistical support for full moderated mediation model.
Gul et al., 2022	Türkiye	Adolescents (12–18, N=197)	Cross-sectional, Path Analysis (AMOS)	Problematic smartphone use (PSU), FoMO	Hostility (measured as psychiatric symptom)	None tested	Gender, Age (as predictors, not moderators)	Hostility significantly increased PSU but decreased FoMO. PSU and FoMO were positively associated. Younger age and male gender predicted higher FoMO.

Table S1. Summary of included studies: mediators, moderators, and key findings

Author(s), Year	Country	Sample (Age, N)	Study design	Social media variable(s)	Aggression type(s)	Mediator(s)	Moderator(s)	Key findings
Hernández et al., 2021	Spain	Adolescents (12–16, N=1763)	Cross-sectional, SEM	Sexting (erotic/pornographic), grooming via social media	Online grooming (sexual aggression)	Personality traits (not mediators, but predictors)	Gender (multi-group SEM)	Disinhibition and narcissism predict grooming and sexting; gender moderates these effects
Jenkins et al., 2024	USA	Middle and high school students, ages 11–18, N = 872	Cross-sectional, quantitative	Witnessing cyberbullying on social media platforms	Cyberbullying	Steps of Bystander Intervention Model (Notice, Interpret, Accept Responsibility, Know How to Help)	Race/ethnicity (students of color vs. White)	Measurement invariance confirmed across groups; White students more likely to report intervention behaviors; students of color followed model steps more strongly once engaged. No significant group differences in first four steps.
Kaakinen et al., 2021	Finland, South Korea, Spain, USA	Adolescents (15–18, N=1451 in Study 1; N=5674 in Study 2)	Cross-sectional, Logistic Regression + Mediation (KHB method)	Online dating apps (e.g., Tinder); social media posting	Online harassment, sexual harassment, peer/adult sexual victimization, other cybercrimes	Risky online routines (e.g., disclosing personal info, seeking strangers)	Gender tested (interaction), not significant	ODA use predicts all forms of victimization; risky online behavior mediates these effects. Cybercrime victimization remained directly associated with ODA use even after mediation. Females and gender-diverse adolescents more

Table S1. Summary of included studies: mediators, moderators, and key findings

Author(s), Year	Country	Sample (Age, N)	Study design	Social media variable(s)	Aggression type(s)	Mediator(s)	Moderator(s)	Key findings
								vulnerable to sexual victimization.
Kee et al., 2024	Malaysia	Youth aged 15–26 (N = 534)	Cross-sectional, SEM (Smart PLS)	Cyberbullying victimization	Cyberbullying (victimization)	Anxiety, Stress, Emotional Exhaustion, Depression	None tested	Cyberbullying positively predicts depression and suicide ideation; anxiety, stress, and exhaustion mediate between cyberbullying and depression, and depression mediates the effect on suicide ideation.
Kim et al., 2024	USA	Early adolescents (11–13, N=316)	Cross-sectional, SEM	Problematic Social Media Use and Conflict (PSMUC)	Cyber-victimization (school-based, via social media)	Social stress (loneliness, exclusion)	None	PSMUC directly and indirectly predicted cyber-victimization via social stress. Social stress significantly mediated the relationship. Model fit was strong. 29% reported cyber-victimization from someone at school.
Laeheem, 2024	Thailand	Youth (18–25, N=340; ~32.6% aged 18–19)	Cross-sectional, SEM	Social media use (≥3 hrs/day), media violence, messaging apps	Cyberbullying (5 forms: gossip, defamation, impersonation, privacy breaches, blocking)	Negative mental traits (e.g., frustration, anxiety)	None tested	Negative upbringing (1.13), personal violence (0.74), media violence (0.64), and negative mental traits (0.17) significantly predicted

Table S1. Summary of included studies: mediators, moderators, and key findings

Author(s), Year	Country	Sample (Age, N)	Study design	Social media variable(s)	Aggression type(s)	Mediator(s)	Moderator(s)	Key findings
								cyberbullying; mental traits partially mediated other effects. SEM explained 92.3% of cyberbullying variance.
Lin et al., 2024	Italy	Early adolescents (11–15, N=773)	Cross-sectional, Serial Mediation (PROCESS Model 6)	Social Media Addiction (BSMAS), Nighttime social media use (NSSMUS)	Aggressive behaviors (overt + relational)	Nighttime social media use, Sleep quality	None tested	SMA predicts aggression directly and indirectly via nighttime use and poor sleep. Serial mediation confirmed: SMA → nighttime use → poor sleep → aggression. All effects significant.
Liu et al., 2023	China	Adolescents (12–15, N=1,006)	Cross-sectional, SEM + Bootstrapped Moderated Mediation	Cybervictimization (social media, messaging apps, games)	Cybervictimization (victimization only)	Depression	School connectedness	Depression mediates the relationship between cybervictimization and NSSI; this indirect path is moderated by school connectedness. The association is stronger for adolescents with low school connectedness.
Madriaza et al., 2025	International (meta-analysis)	55 studies, 101 effect sizes (n ranges: 69–6829)	Systematic Review + Meta-analysis	Exposure to hate in online media (social networks, forums, etc.)	Online hate speech perpetration, offline violent behavior, online victimization	None tested	Some moderators examined (platform,	Exposure to online hate predicts offline violence (d = 0.47), online hate speech (d = 0.36), and online victimization (d =

Table S1. Summary of included studies: mediators, moderators, and key findings

Author(s), Year	Country	Sample (Age, N)	Study design	Social media variable(s)	Aggression type(s)	Mediator(s)	Moderator(s)	Key findings
							sample characteristics)	0.72); psychological effects include depression and reduced life satisfaction. No evidence that exposure leads to resistance or rejection of hate content.
Mardianto et al., 2023	Indonesia	High school students, N = 1118, ages not precisely specified but adolescent	Quantitative, SEM (Structural Equation Modeling)	Cyber aggression on social media (general usage by students)	Cyber aggression	Prejudice	None	Threat perception and school climate predicted cyber aggression. Prejudice significantly mediated both relationships. Higher threat perception and poorer school climate increased prejudice, which in turn increased cyber aggression.
Marinoni et al., 2024	Italy	Adolescents (11–19, N=4338)	Cross-sectional, Moderated Mediation (PROCESS Model 7)	Social media use, online gaming	Cyberbullying (perpetration) and cybervictimization	Time spent online	Sex	Social media linked to cyberbullying (girls), online gaming to cybervictimization (boys); time online mediates both; sex moderates effects. Cyberbullying risk increased with more time online, especially

Table S1. Summary of included studies: mediators, moderators, and key findings

Author(s), Year	Country	Sample (Age, N)	Study design	Social media variable(s)	Aggression type(s)	Mediator(s)	Moderator(s)	Key findings
								among girls using social media and boys playing games.
Martínez-Ferrer et al., 2021	Spain	Adolescents (12–18, N=2011)	Cross-sectional, Moderated Mediation (PROCESS Model 59)	Problematic social networking site use (PSNSU)	Cyberbullying, Cybervictimization, Offline victimization	PSNSU	Gender	PSNSU mediates link between cyber/offline victimization and cyberbullying. Victimized girls show higher PSNSU; boys show more cyberbullying. Gender moderates direct effects, but not the indirect (mediated) effects.
Maurya et al., 2023	India	Adolescents and young adults (12–23, N=16,292; 84% ≤19)	Cross-sectional, SEM	Cyber victimization (harassment via phone or internet)	Cyber victimization	Self-efficacy, Parental communication	None	Cyber victimization significantly predicted depressive symptoms. Self-efficacy and parental communication partially mediated the relationship. Stronger parent–child communication and higher self-efficacy reduced depression linked to victimization.
Q. Pan et al., 2024	Hong Kong	Adolescents (12–18, N=1,239)	Cross-sectional, SEM	Online risk exposure (including	Cyberbullying exposure	Digital resilience components	Gender, SES, digital literacy,	Recovery and learning from cyberbullying

Table S1. Summary of included studies: mediators, moderators, and key findings

Author(s), Year	Country	Sample (Age, N)	Study design	Social media variable(s)	Aggression type(s)	Mediator(s)	Moderator(s)	Key findings
				cyberbullying); coping strategies		(recovery, learning, coping strategies)	parenting, school curricula	experiences enhance wellbeing; non-productive coping reduces wellbeing; DR mediates risk–wellbeing relationship; school and parent factors influence DR development
Y. Pan et al., 2024	Malaysia	Adolescents (12–18, N=595)	Cross-sectional, Path Analysis (PROCESS)	Game addiction (via social media recruitment)	Aggression (verbal, physical, anger, hostility – BPAQ)	Narcissism, Self-control	None tested	Game addiction significantly predicts aggression (β = 0.777). Narcissism (β = 0.347) and self-control (β = 0.209) both partially mediate the relationship. Lower self-control and higher narcissism explain the aggression pathway.
Peprah et al., 2024	35 high-income countries	Adolescents (11–16.5, N=142,298)	Cross-sectional, path analysis with bootstrapping	Cyberbullying victimization, Problematic Social Media Use (PSMU)	Cyberbullying victimization (online)	Problematic Social Media Use (PSMU)	None (moderation by PSMU also tested)	Cyberbullying victimization significantly predicted psychosomatic complaints. PSMU partially mediated the relationship (12% of total effect). Moderation by PSMU also observed.

Table S1. Summary of included studies: mediators, moderators, and key findings

Author(s), Year	Country	Sample (Age, N)	Study design	Social media variable(s)	Aggression type(s)	Mediator(s)	Moderator(s)	Key findings
Piccardi et al., 2023	Italy	Adolescents (14–20, N=366)	Cross-sectional, Mediation (SPSS PROCESS)	Online vulnerability via social media (exposure to violent, harmful content)	Online vulnerability (victimization-focused)	Moral disengagement (specifically Displacement of Responsibility)	None tested	Gender predicted online vulnerability; boys used more MD mechanisms; Displacement of Responsibility mediated the gender-vulnerability relationship; older age associated with higher vulnerability.
Qiu et al., 2024	China	College students (Mage = 20.74, N = 1,259)	Cross-sectional, Moderated Mediation (PROCESS Model 59)	Antisocial media exposure (e.g., violence, hate content)	Malicious online trolling (aggression-like behavior)	Hostile attribution bias	Empathy	Antisocial media exposure predicted trolling directly and indirectly via hostile attribution bias. Empathy weakened all direct and indirect effects. Moderated mediation confirmed.
Ramírez et al., 2021	Chile	Children and early adolescents (9–12, N=2,440)	Cross-sectional, SEM	Cellphone use, video games, online exposure (e.g., violent content, strangers)	Cyberbullying (victimization, perpetration, bystander)	Sleep deprivation (between tech use and GPA)	None tested	Cyberbullying victimization negatively associated with GPA and life satisfaction. Sleep deprivation mediated the link between cellphone/video game use and GPA. 42% played with strangers;

Table S1. Summary of included studies: mediators, moderators, and key findings

Author(s), Year	Country	Sample (Age, N)	Study design	Social media variable(s)	Aggression type(s)	Mediator(s)	Moderator(s)	Key findings
								9.7% experienced cyberbullying.
Reed et al., 2021	USA	Adolescents (Mean age = 16.4, N = 703)	Cross-sectional, SEM	Time on social media, digital communication with dating partners	Digital dating abuse (monitoring/control, direct aggression, sexual coercion)	None	Gender (model stratified by sex)	SGDBs predicted DDA perpetration: girls → monitoring/control & sexual coercion; boys → direct aggression & sexual coercion. Digital behaviors aligned with traditional gender roles.
Shahnawaz et al., 2020	India	Adolescents aged 16–19, N = 207	Cross-sectional, quantitative	Cyber aggression on social media (general)	Cyber aggression	None	Moral Identity (Internalization, Symbolization)	Sadism predicted cyber aggression. Moral identity (internalization) moderated the relationship—only high moral identity reduced aggression. Symbolization had no significant moderating effect. Surprisingly, internalization also predicted aggression positively.
Soriano-Molina et al., 2025	International (meta-analysis)	Adolescents (mean age ≈ 14.6, N=303,823)	Meta-analysis (33 studies)	Generalized internet addiction (includes social media use)	Aggressiveness (self-report, externalizing behavior)	None tested	Age, gender, continent, study design (no significant moderators)	Internet addiction showed the strongest positive correlation with aggressiveness (r = 0.391); IA also linked to

Table S1. Summary of included studies: mediators, moderators, and key findings

Author(s), Year	Country	Sample (Age, N)	Study design	Social media variable(s)	Aggression type(s)	Mediator(s)	Moderator(s)	Key findings
								depression, anxiety, and low well-being. Moderators like age, gender, and geography had no significant effect.
Strickland et al., 2023	USA	Adolescents (11–18, N=1,960 in relationships)	Cross-sectional, Hierarchical Regression	Cyber dating abuse (texts, social media threats, pressure for nudes, monitoring)	Cyber dating abuse (victimization)	None	Parent–child closeness, communication, shared activities	Closeness moderated the CDA–mental health link: higher closeness reduced depression/anxiety. Communication and activities did not moderate effects. CDA predicted worse mental health; girls more affected than boys.
Tamarit et al., 2021	Spain	Adolescents (12–16, N=1,763)	Cross-sectional, SEM	Internet/social media addiction (ERA-RSI); social media use, geek behavior	Online sexual victimization: erotic/coercive sextortion, grooming	Body self-esteem (body satisfaction, physical attractiveness), Erotic & Pornographic sexting	None tested	Internet addiction predicts online victimization. Body satisfaction and sexting partially mediate the link between addiction and grooming/sextortion. Geek behavior and sexting (especially pornographic) are key

Table S1. Summary of included studies: mediators, moderators, and key findings

Author(s), Year	Country	Sample (Age, N)	Study design	Social media variable(s)	Aggression type(s)	Mediator(s)	Moderator(s)	Key findings
								indirect pathways to grooming.
Tan, 2023	Japan	Gen Z (15–25, N = 533)	Cross-sectional, SEM	False self, POSI, CIU, Online Disinhibition	Cyberaggression	Moral disengagement, Cyberaggression (also serial mediation)	None tested	False self and online disinhibition predicted moral disengagement and cyberaggression; these mediated the effects on social withdrawal. Serial mediation path (false self → MD → CA → SW) also significant.
Virat et al., 2025	France	Adolescents (10–21, N=428)	Cross-sectional, Mediation (Bootstrap via Jamovi)	Recruitment via social media; no specific platforms measured	Delinquent intentions (non-violent + violent)	Attitudes toward justice system, Peers' legal attitudes	None tested	Lower societal mattering predicted higher delinquent intentions. This link was partially mediated by peers' attitudes toward law and personal trust in justice. Effects remained after controlling for past delinquency.
Wang et al., 2023	China	Early adolescents, Mage = 13.26, N = 213	Cross-sectional, path analysis	Attention-seeking and impulsive social media use	Cyberbullying	Attention-seeking, impulsivity	Loneliness	Narcissism predicted both cyberbullying and online prosocial behaviors via attention-seeking (not impulsivity). Loneliness

Table S1. Summary of included studies: mediators, moderators, and key findings

Author(s), Year	Country	Sample (Age, N)	Study design	Social media variable(s)	Aggression type(s)	Mediator(s)	Moderator(s)	Key findings
								moderated the effect of narcissism on attention-seeking, strengthening its role in cyberbullying and prosociality.
Yusuf et al., 2021	Malaysia	Adolescents (13–18, N=430)	Cross-sectional, SEM (AMOS)	Internet exposure (e.g., violent, antisocial media content); smartphone/social media use	Cyber aggression-victimization (visual, verbal, exclusion, impersonation)	None tested	None tested	Internet exposure was the only significant predictor ($\beta = .445$, $p < .001$); parental and peer attachment were not significant. Youth spent up to 21+ hrs online on weekends, primarily on social media.
Y. Zhang et al., 2021	China	Adolescents (12–17, N=1,204)	Cross-sectional, Moderated Moderation (PROCESS Model 3)	Social media cyberbullying during COVID-19 + floods	Cyberbullying perpetration	None	Parent–child relationships, Negotiable fate (cultural belief)	Psychological distress predicts cyberbullying; effect weakened by high-quality PCRs and high negotiable fate. When negotiable fate is low, distress strongly predicts cyberbullying regardless of PCRs.
Z. Zhang et al., 2022	China	Adolescents (11–20, N=501)	Cross-sectional, Moderated Mediation	Cyber aggression via online games, social media (instrumental aggression)	Cyber Aggression (verbal insults, scolding in digital contexts)	Moral Disengagement	Gender	All Dark Triad traits predicted CA; MD partially mediated these effects. Contrary to predictions, DT traits

Table S1. Summary of included studies: mediators, moderators, and key findings

Author(s), Year	Country	Sample (Age, N)	Study design	Social media variable(s)	Aggression type(s)	Mediator(s)	Moderator(s)	Key findings
			(PROCESS Models 4 & 5)					were more strongly associated with CA among female adolescents than males.
Zimmer-Gembeck et al., 2021	Australia	Adolescents (15–19, N=650)	Longitudinal (1 year), SEM	Time spent on social media, appearance-related cyber-victimization	Cyber-victimization (appearance-related)	None tested	Gender (moderator of symptom stability only)	Face-to-face victimization (not cyber) uniquely predicted increases in offline appearance anxiety and online appearance preoccupation. Strong bidirectional link found between the two outcomes. Cyber-victimization was correlated but not predictive over time. Gender moderated symptom stability (higher in girls).
