# Supplementary material for: Mediating and Moderating Mechanisms in the Relationship Between Social Media Use and Adolescent Aggression: A Scoping Review of Quantitative Evidence
Source: Eur J Investig Health Psychol Educ. 2025 Jun 3;15(6):98. doi: 10.3390/ejihpe15060098 (PMC12192510; doi:10.3390/ejihpe15060098)
Supplement: Supplementary file 1 [file ejihpe-15-00098-s001.zip › Supplementary Table S2.pdf]

Table S2. Study quality assessment using the Mixed Methods Appraisal Tool (MMAT)

Study	MMAT Domain Rating				
	**Participants representative**	**Appropriate measurements**	**Complete outcome data**	**Confounders accounted**	**Nonresponse bias low**
Achuthan et al., 2022	Yes	Yes	Yes	Yes	Unclear
Aljasir & Alsebaei, 2022	Yes	Yes	Yes	Yes	Unclear
Alsebaei & Aljasir, 2024	Yes	Yes	Yes	Partially	Unclear
Bakan Kıraç et al., 2024	Yes	Yes	Yes	Partially	Unclear
Bedrosova et al., 2025	Yes	Yes	Yes	Yes	Yes
Cebollero-Salinas et al., 2022	Yes	Yes	Yes	Yes	Yes
Q. Chen et al., 2025	Yes	Yes	Yes	Yes	Yes
Colella et al., 2024	Yes	Yes	Yes	Yes	Unclear
Colella et al., 2025	Yes	Yes	Yes	Yes	Unclear
Doğrusever & Bilgin, 2025	Yes	Yes	Yes	Yes	Yes
Ferreira et al., 2021	Yes	Yes	Yes	Yes	Yes
Geng et al., 2021	Yes	Yes	Yes	Yes	Yes
Gul et al., 2022	Yes	Yes	Yes	Yes	Yes
Hernández et al., 2021	Yes	Yes	Yes	Yes	Yes
Jenkins et al., 2024	Yes	Yes	Yes	Yes	Yes
Kaakinen et al., 2021	Yes	Yes	Yes	Yes	Yes
Kee et al., 2024	Yes	Yes	Yes	Partially	Unclear
Kim et al., 2024	Yes	Yes	Yes	Partially	Unclear
Laeheem, 2024	Yes	Yes	Yes	Partially	Yes
Lin et al., 2024	Yes	Yes	Yes	Partially	Yes
Liu et al., 2023	Yes	Yes	Yes	Yes	Yes
Madriaza et al., 2025	Yes	Yes	Yes	Yes	Yes
Mardianto et al., 2023	Yes	Yes	Yes	Partially	Yes
Marinoni et al., 2024	Yes	Yes	Yes	Partially	Yes
Martínez-Ferrer et al., 2021	Yes	Yes	Yes	Yes	Yes
Maurya et al., 2023	Yes	Yes	Yes	Yes	Yes
Q. Pan et al., 2024	Yes	Yes	Yes	Yes	Yes
Y. Pan et al., 2024	Partially	Yes	Yes	Partially	Unclear

Peprah et al., 2024	Yes	Yes	Yes	Yes	Yes
Piccardi et al., 2023	Yes	Yes	Yes	Yes	Yes
Qiu et al., 2024	Yes	Yes	Yes	Yes	Yes
Ramírez et al., 2021	Yes	Yes	Yes	Yes	Yes
Reed et al., 2021	Yes	Yes	Yes	Yes	Yes
Shahnawaz et al., 2020	Yes	Yes	Yes	Partially	Yes
Soriano-Molina et al., 2025	Yes	Yes	Yes	Yes	Yes
Strickland et al., 2023	Yes	Yes	Yes	Yes	Yes
Tamarit et al., 2021	Yes	Yes	Yes	Partially	Yes
Tan, 2023	Partially	Yes	Yes	Partially	Yes
Virat et al., 2025	Yes	Yes	Yes	Yes	Yes
Wang et al., 2023	Yes	Yes	Yes	Yes	Yes
Yusuf et al., 2021	Yes	Yes	Yes	Partially	Yes
Y. Zhang et al., 2021	Yes	Yes	Yes	Yes	Yes
Z. Zhang et al., 2022	Yes	Yes	Yes	Yes	Yes
Zimmer-Gembeck et al., 2021	Yes	Yes	Yes	Yes	Yes
